# Nitric Oxide as a Remedy against Oxidative Damages in Apple Seeds Undergoing Accelerated Ageing

**DOI:** 10.3390/antiox11010070

**Published:** 2021-12-28

**Authors:** Katarzyna Ciacka, Marcin Tyminski, Agnieszka Gniazdowska, Urszula Krasuska

**Affiliations:** Department of Plant Physiology, Institute of Biology, Warsaw University of Life Sciences, Nowoursynowska 159, 02-776 Warsaw, Poland; marcin_tyminski@sggw.edu.pl (M.T.); agnieszka_gniazdowska@sggw.edu.pl (A.G.); urszula_krasuska@sggw.edu.pl (U.K.)

**Keywords:** accelerated ageing, control deterioration treatment, reactive nitrogen species, reactive oxygen species, seed ageing, seed germination, seed vigour

## Abstract

Seed ageing is associated with a high concentration of reactive oxygen species (ROS). Apple (*Malus domestica* Borkh.) seeds belong to the orthodox type. Due to a deep dormancy, they may be stored in dry condition at 5 °C for a long time, without viability loss. In the laboratory, artificial ageing of apple seeds is performed by imbibition in wet sand at warm temperature (33 °C). The aim of the work was to study nitric oxide (NO) as a seed vigour preservation agent. Embryos isolated from apple seeds subjected to accelerated ageing for 7, 14, 21 or 40 days were fumigated with NO. Embryo quality was estimated by TTC and MDA tests. ROS level was confirmed by NBT staining. We analysed the alteration in transcript levels of *CAT*, *SOD* and *POX*. NO fumigation of embryos of seeds aged for 21 days stimulated germination and increased ROS level which correlated to the elevated expression of *RBOH*. The increased total antioxidant capacity after NO fumigation was accompanied by the increased transcript levels of genes encoding enzymatic antioxidants, that could protect against ROS overaccumulation. Moreover, post-aged NO application diminished the nitro-oxidative modification of RNA, proving NO action as a remedy in oxidative remodelling after seeds ageing.

## 1. Introduction

Viable seeds, as the only mobile form of higher plants, allow the dispersal of species on Earth. The high quality of seeds determines further plants growth and development. Thus, seed longevity, defined as the total time span during which seed remains viable, is the primary issue for ecology, agronomy and economy [1,2]. Seed longevity depends mostly on genetic material and differs for various species [3]. Naturally, seeds evolved to avoid inconvenient environmental conditions. This ability is supported by dormancy state [4,5]. However, even deeply dormant seeds are not immortal, they undergo ageing during storage, also under conditions of low temperature and low humidity [1,2]. Inadequate storage conditions accelerate cellular deterioration and promote seed ageing. An increased membrane permeability, progression of DNA fragmentation and proteomic damage, accompanied by a lower scavenging capacity of the antioxidant system lead to loss of seed viability [6,7]. Additional markers of seed viability are late embryogenesis abundant protein or heat shock protein, or changes in stored mRNAs level [6,8,9,10]. Typical effects of decreased seed viability are the reduced germination rate, the lower number of developed seedlings and the increased amount of seedlings with morphological anomalies [11].

In the laboratory practice, to obtain lots of seeds of equally decreased viability, artificial ageing protocols are used by the application of high temperature (25–45 °C) and high humidity (75–100%) [7,12]. The accelerated ageing rate parameter may be used as a good predictor of the capacity of seed storability [13]. Accelerated ageing and controlled deterioration treatment (CDT) are the most commonly used methods in the seeds laboratory practice. The difference between CDT and accelerated ageing is that during CDT seeds are imbibed to a precise water content prior to the warm temperature treatment [6]. Among compounds involved in the regulation of ageing in living organisms, reactive oxygen species (ROS) are recognised [14,15]. A cellular oxidative imbalance is the basis of the concept of the free radical theory of ageing. Free radicals include superoxide anion (O_2_^•−^) and hydroxyl radical (^•^OH) [16] of high biochemical reactivity. Non-radical hydrogen peroxide (H_2_O_2_) at high concentration is also involved in cellular damage. ROS have the ability to react with nucleic acids, proteins, lipids and sugars [16,17,18]. ROS attack on cellular membranes results in the increase in the malondialdehyde (MDA) level, the characteristic marker of loss of seed vigour [7]. The reaction of free radicals with proline, arginine, lysine, asparagine or threonine residues and the incorporation of reactive carbonyl derivatives by interaction with cysteine, histidine or lysine, lead to the formation of carbonyl groups (CO) in proteins [18]. The modification drives proteins to degradation. This strategy (degradation of carbonylated proteins) is desirable as it allows to avoid the formation of dysfunctional aggregates [14]. In plants, the high content of CO groups in proteins is characteristic for biotic and abiotic stress responses [19], for dormant [20] or aged [8] seeds. Under physiological conditions, the formation of CO groups in proteins is irreversible. Thus, the level of carbonylated proteins may serve as the oxidative stress marker [14]. However, the highest level of carbonylated proteins was detected in the axes of vigorous dormant apple (*Malus domestica* Borkh.) embryos [20]. Besides proteins, ROS-dependent disruption of nucleic acids was noticed in aged seeds [21], and RNA is more prone to damage caused by ROS than double-stranded DNA ([22] and references herein). During seed maturation, long-lived mRNAs are stored, and translated into proteins at the early phases of germination [10]. The selective oxidation of mRNA was related to after-ripening of sunflower seeds [23]. The transcript oxidation was low when seeds were dormant and increased when dormancy was lost. It suggests the role of such modified mRNA in seed transition from dormant to non-dormant state.

ROS level is under the control of proteins which participate in their synthesis or detoxification [16]. A nicotinamide adenine dinucleotide phosphate oxidase (NADPH-oxidase, also known as the burst oxidase homologue—RBOH) localized in the plasma membrane, is an enzyme, responsible for O_2_^•−^ generation in apoplastic space. In the *Arabidopsis* genome ten members encoding RBOH: *AtRBOHA-J* are present. As was demonstrated, *AtRBOHB* was positively implicated in seeds after-ripening [24]. Enzymatic antioxidants are superoxide dismutases (SODs), catalases (CATs) and peroxidases [25]. Peroxidases (POXs) of Class III, haem-containing glycoproteins play a double role in ROS metabolism, as scavengers or producers. The high activity of these enzymes has been documented for apple embryos during nitric oxide (NO) dependent dormancy breakage [26].

Nitric oxide (NO), and its derivatives—reactive nitrogen species (RNS) are the regulatory molecules in seeds, as described in detail by the model of the “nitrosative door”. NO acts as an intracellular regulator of seed dormancy/germination [27]. An implication of NO in seed ageing was proposed [15], as the influence of this compound on plant senescence has been demonstrated [28,29]. RNS participate in the modification of proteins, fatty acids and nucleic acids [30,31,32]. One of the RNS-dependent posttranslational protein modifications is nitration [33]. The process of tyrosine (Tyr) nitration is irreversible under physiological conditions [33]. Similarly, to the level of CO groups in proteins, the 3-nitro-Tyr (3-NT) content is considered as a marker of nitro-oxidative stress. Protein nitration is a two-step reaction initiated in the presence of peroxynitrite (ONOO^−^), generated from O_2_^•−^ and NO [34]. The interaction between ONOO^−^ and nucleic acid, resulting in 8-nitroguanine (8-NO_2_-G) formation is also known but still not well documented in plants [31,35,36]. Dormancy loss of apple embryos is accompanied by the decrease in total nitrated protein content in embryonic axes during germination [37], while the increase in nitrated RNA level was detected [31].

Apple seeds belong to the *orthodox* type, that tolerate dehydration during maturation, are resistant to adverse environmental factors, and age slowly. It is due to the glassy state formation, that lowers the rate of browning reactions, enhances enzymes stability, and preserves proteins structure and function [38]. Mature apple seeds are deeply dormant [39]. Their dormancy is removed by several weeks long cold stratification (seeds placement in wetted sand at 4–5 °C). Embryos isolated from cold stratified seeds germinate fast and develop typical seedlings [11,39]. Contrary, warm stratification of seeds (in wetted sand at 25 °C) did not break embryonic dormancy, as a lower percentage of germinated embryos, and a higher number of seedlings with morphological anomalies have been demonstrated [11]. The experimental environment of prolonged warm stratification, used by us previously, corresponds to the conditions of accelerated ageing. It has been detected the lower NO emission from embryonic axes isolated from warm stratified apple seeds compared to the NO emission from embryonic axes of cold stratified seeds [11]. Thus, we assume that the beneficial effect of NO could be also observed on the preservation of seed viability and regulation of seeds ageing.

The aim of this work was to investigate the possible implication of NO in the maintenance of *orthodox*-type seed viability. We examined the impact of the duration of apple seeds artificial ageing (7, 14, 21 and 40 days) and the role of NO in mitigation of the negative effects of ageing. We have checked if short-term fumigation with NO of the embryos isolated from seeds subjected to accelerated ageing influences basic markers of seed viability loss: MDA, ROS level and total antioxidant capacity. To provide a new insight into the role of NO in seed longevity preservation we have measured the content of nitro-oxidatively modified proteins and nucleic acids and the transcript levels of genes encoding enzymatic antioxidants. The performed experiments allow us to formulate the conclusion that NO, as a post-ageing factor, may be in some circumstances used as the remedy to re-establish decreasing viability of the aged seeds.

## 2. Materials and Methods

### 2.1. Plant Material

The experiments were carried out on apple (*Malus domestica* Borkh. cv. Antonówka) seeds subjected to artificial ageing by application of the accelerated ageing protocol. The ripened apple fruits were obtained from the WIKPOL fruit producer (Dąbrowa, Poland). The dormant seeds, after isolation from the fruits, were dried at room temperature and stored in dark glass containers at 5 °C for further procedures.

Accelerated ageing procedure: the dormant seeds were mixed with sterile quartz sand at 65% humidity and kept at 33 °C in darkness for 7, 14, 21 and 40 days. Every three days, seeds and sand were well mixed to avoid oxygen deprivation. The constant sand humidity was kept during the entire period of the procedure. Before the analyses, seeds were removed from the sand, rinsed with water, and the embryos were isolated from seed coats.

NO treatment: just after removal of the seed coats from artificially aged seeds, a half part of the embryos was shortly (3 h) fumigated with NO released from a solution of acidified nitrite (14.5 mM NaNO_2_, 0.2 M HCl) placed in a glass container tightly closed for 3 h of the NO treatment. After treatment, the embryos were washed in distilled water.

The non-treated embryos (the control, embryos isolated from seeds subjected only to accelerated ageing) and embryos isolated from seeds subjected to ageing and after short fumigation with NO (NO treatment) were placed in glass Petri dishes on filter paper moistened with distilled water for 48 h in a growth chamber (Sanyo MLR-35OH) at 25/20 °C day/night, a 12/12 h photoperiod, the light intensity of 100 μmol PAR m^−2^ s^−1^ and used for further analyses. 

**Figure 1:**
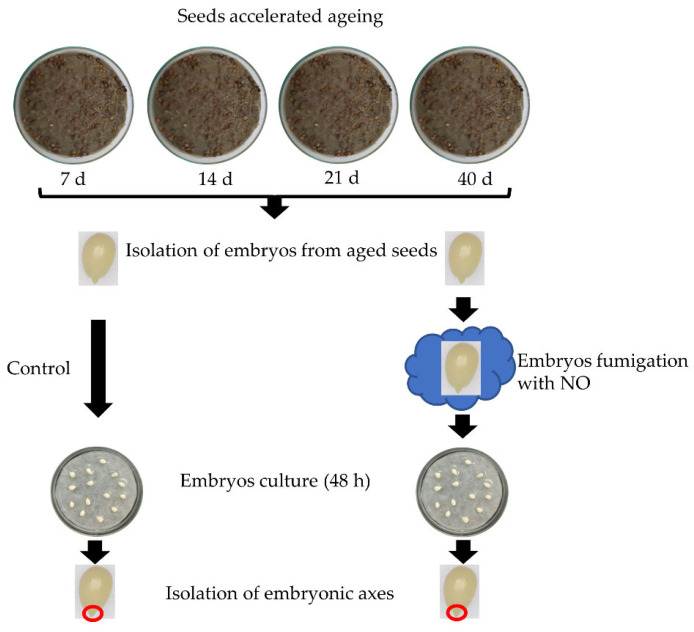
The scheme of the experimental model. Apple seeds were aged for 7, 14, 21 or 40 days on Petri dishes filled with moisture sand at 33 °C. After accelerated ageing of the seeds, embryos were isolated and part of them was fumigated with NO. Embryos non-treated with NO were used as a control. Embryos (control and NO treated) were cultured for 48 h and the embryonic axes were isolated and used for biochemical and transcriptomic analysis. The germination rate of the embryos and morphology of developing seedlings were determined after 7 days of the culture.

The experimental model was presented in Figure 1.

### 2.2. Germination Tests

The apple embryos isolated from (1) artificially aged seeds for 7, 14, 21 and 40 days, non-treated with NO (the control) and (2) embryos isolated from artificially aged seeds for 7, 14, 21 and 40 days shortly fumigated with NO as described above, were used for germination tests, performed in glass Petri dishes (15 embryos per dish, three dishes per treatment) on filter paper moistened with distilled water. The embryos (control and treated with NO) germinated for 7 days in the growth chamber (Sanyo MLR-35OH) at 25/20 °C day/night, a 12/12 h photoperiod, and the light intensity of 100 μmol PAR m^−2^ s^−1^. The termination of germination sensu stricto was defined by a characteristic gravitropic bending of an embryonic axis. Germination tests were performed in three independent experiments.

### 2.3. Cell Viability Test

A cell viability test was performed by staining the apple embryos with 2,3,5-triphenyltetrazolium chloride (TTC) (Sigma-Aldrich, St. Louis, MO, USA). Red coloration of the tissue visualizes dehydrogenase activity. The isolated embryos were briefly washed twice in distilled water, and placed in the solution of 2% (*w*/*v*) TTC dissolved in 0.9% (*w*/*v*) sodium chloride (NaCl) with the addition of 2 mM dimethyl sulfoxide (DMSO). Staining was carried out for 20 min at room temperature in darkness. After incubation in TTC, the embryos were washed with distilled water and photographed (TAGARNO FHD TREND). The viability of cells was estimated according to colour appearance. Experiments were performed in three independent repetitions and representative data were shown.

### 2.4. In Situ O_2_^•−^ Localization 

Localization of O_2_^•−^ in the apple embryos was performed using nitroblue tetrazolium (NBT) staining which reacts with O_2_^•−^ and forms dark blue formazan. The embryos were placed in 2 mM NBT solution: NBT dissolved in 10 mM Tris-HCl pH 7.4 with 2 mM DMSO. After incubation (20 min) the embryos were transferred to chloral hydrate solution and incubated at 4 °C overnight to remove phenolics. After washing with distilled water, the embryos were photographed (TAGARNO FHD TREND). Experiments were performed in four independent repetitions and representative data were shown.

### 2.5. Visualisation of MDA 

Visualization of aldehyde groups, including MDA formed during lipid peroxidation was performed using Schiff reagent [40]. The apple embryos, after 48 h of the culture were washed in distilled water and placed in a container with Schiff reagent for 20 min at room temperature in darkness. After incubation, the embryos were washed in distilled water and placed in the solution of 0.5% (*w*/*v*) sodium metabisulfite (Chempur 118066503) in 0.05 M HCl for 2 min. Next, the embryos were washed with distilled water and photographed (TAGARNO FHD TREND). Experiments were performed in three independent repetitions and representative data were shown.

### 2.6. Analysis of DNA Concentration 

DNA was isolated from the embryonic axes using Plant DNA Mini Kit (Syngen), following the manufacturer’s guideline. The embryonic axes (50 mg) were ground in liquid nitrogen, and the powder was transferred into the Eppendorf tube and mixed well with 400 µL of lysis buffer (DLR1) and 8 µL of 5% (*w*/*v*) RNAse A solution. The sample was incubated for 10 min at 65 °C, using a thermal shaker (VWR Thermal Shake Lite). Next, 130 µL of another lysis buffer (DLR3) was added and the sample was incubated for 5 min at 4 °C. The lysate was transferred into the KL column, added to the kit, and centrifuged at 4 °C for 3 min, 20,000× *g*. The obtained filtrate was mixed with DWR buffer (1:1.5 ratio) in the new Eppendorf tube. The mixture (700 µL) was transferred into the DR column. Then, the column was centrifuged at 4 °C for 2 min, 20,000× *g* and the filtrate was removed. These steps were repeated using the same DR column till all mixture was filtrated. Thereafter, 500 µL of washing buffer (DP1) was added into the DR column. After centrifugation at 4 °C for 1 min, 20,000× *g*, 750 µL of another washing buffer (DP2) was added and centrifuged again at the same conditions. When the filtrate was removed, the column was placed in the new Eppendorf tube and 50 µL of elution buffer (DE) heated to 65 °C was added. The sample was incubated at 37 °C for 10 min and centrifuged for 2 min at 22 °C, 20,000× *g*. The elution step was repeated, adding 40 µL of DE buffer. 

The quality and quantity of DNA were measured using NANODROP 2000 Spectrophotometer (Thermo Scientific, Waltham, MA, USA). The content of DNA was expressed as ng DNA mg^−1^ fresh weight (FW). Experiments were performed in three–four independent biological repetitions with three repetitions in each.

### 2.7. Measurement of Total Antioxidant Capacity

The total antioxidant capacity test was performed using 2,2-diphenyl-1-picrylhydrazyl (DPPH) (Sigma-Aldrich D9132) reduction assay [41]. The apple embryonic axes (40 mg) were homogenized in 0.4 mL of 80% (*v*/*v*) methanol on ice and incubated in an ultrasonic bath at 4 °C for 5 min. After centrifugation (10 min at 4 °C, 20,000× *g*), 7.5 μL of supernatant was added to 292.5 μL of 60 μM DPPH solution in 100% methanol and incubated at room temperature for 15 min in darkness. The concentration of reduced DPPH was measured at 517 nm using a microplate reader (Sunrise, Tecan, Männedorf, Switzerland). Total antioxidant capacity was expressed as reduction of DPPH calculated using the equation: [(A_0_ − A_s_)/A_0_] × 100%, where A_0_ is the absorbance of a blank and A_s_ is the absorbance of the sample. Experiments were performed in three to four independent replicates with three repetitions in each.

### 2.8. Quantitative Measurement of Carbonylated Proteins

Carbonyl groups (CO) in proteins isolated from the apple embryonic axes were measured using enzyme-linked immune sorbent assay (ELISA) according to Buss et al. [42] with slight modifications described by [43]. The isolated embryonic axes (50 mg) were homogenized in 0.5 mL of the mixture: 0.1 M Tris-HCl, pH 7.0, 2% (*w*/*v*) PVPP, 1 mM DTT, 1% (*v*/*v*) protease inhibitor cocktail (Sigma-Aldrich P9599), and 10% (*v*/*v*) glycerol in the ice bath, and shortly mixed in vortex. After centrifugation at 20,000× *g* 15 min at 4 °C, the content of protein was measured using Bradford (1976) reagent (Supelco B6916). Isolated proteins with CO groups were labelled with 10 mM 2,4-dinitrophenylhydrazine (DNPH Sigma-Aldrich) dissolved in 10 mM DMSO for 30 min at 35 °C in darkness. 

Oxidation of bovine serum albumin (BSA): Reduction of fatty acid-free BSA (Sigma-Aldrich) was performed using sodium dithionite (2 mM) as described in Krasuska et al. [43]. Solution of reduced BSA was loaded into Pierce concentrator PES 3K (Thermo Scientific) for desalting. Oxidation of reduced BSA was performed using 1 mL of 3% (*v*/*v*) H_2_O_2_ with the addition of 0.1% sodium perchlorate. Oxidized BSA was labelled with DNPH. Blank probes of BSA were incubated in 10 mM DMSO without the addition of DNPH. BSA was precipitated using 20% (*w*/*v*) trichloracetic acid (TCA, EUROCHEM 200-927-2) for 20 min at room temperature, centrifuged (20,000× *g*; 10 min), and dissolved in water. The pH of BSA solution was adjusted to 9.0 with 1 M KOH. The concentration of CO groups in BSA was spectrophotometrically measured at 375 nm, and calculated using the extinction coefficient ε = 22 mM^−1^ cm^−1^ [43].

Triplicate of each sample (200 µL) or oxidized BSA (the positive control) were added to wells of Nunc Immuno Plate Maxisorp, and after incubation at 4 °C overnight, the plate was blocked with 0.1% (*w*/*v*) reduced BSA in Tris-buffered saline (TBS). After washing steps with TBST, monoclonal antibodies (Monoclonal Anti-dinitrophenyl (DNP) antibodies, A2831 Sigma-Aldrich) conjugated with alkaline phosphatase (dilution 1:25,000) in TBST (TBS with 0.05% (*v*/*v*) Tween-20) were added. As a substrate for alkaline phosphatase *p*-nitrophenyl phosphate sodium (*p*NPP) was used. Absorbance was read at 405 nm with referential wave 605 nm in the microplate reader (Sunrise, Tecan). Measurements were performed in three independent experiments with three repetitions in each. The results were expressed as nmol of CO groups mg^−1^ protein.

### 2.9. Quantitative Measurement of Nitrated Proteins

The residues of 3-NT in proteins were quantified using the ELISA method as described by [37]. The isolated embryonic axes (50 mg) were homogenized in 0.1 M HEPES-KOH, pH 7.0, 2% (*w*/*v*) PVPP, 2 mM DTT, 1% (*v*/*v*) protease inhibitor cocktail (Sigma Aldrich) and 10% (*v*/*v*) glycerol in an ice bath. After short mixing in a vortex, probes were centrifuged at 20,000× *g* for 15 min, 4 °C. The supernatants were collected for the determination of protein concentration (Bradford assay) and 3-NT level. 

Nitration of BSA: Positive control was prepared from fatty acid-free BSA dissolved in TBS and incubated with NaNO_2_ (1 mM) acidified with 0.2 M HCl in the presence of 0.1 mM NaHCO_3_ for 30 min at 37 °C, in darkness. Then BSA was precipitated with 20% (*w*/*v*) TCA for 20 min at room temperature, centrifuged (20,000× *g*, 10 min), and dissolved in water. After adjusting the pH of the solution to 9.0 with 1 M KOH, the 3-NT content in nitrated BSA was determined at 438 nm, and calculated using the extinction coefficient ε = 4.3 mM^−1^ cm^−1^.

Nunc Immuno Plate Maxisorp (Sigma) was coated with samples (200 µL) or nitrated BSA and incubated at 4 °C overnight. Next, the plate was washed three times with PBS, blocked with 0.1% (*w*/*v*) gelatine in TBS (250 µL per well) (1.5 h at 37 °C in darkness), and washed again three times with TBST. Monoclonal primary antibodies (Monoclonal Anti-3-NT antibodies, Sigma -Aldrich) were added (dilution 1:1000) and incubated for 1 h at 37 °C in darkness. After incubation, the plate was washed three times with TBST and covered with secondary antibodies (anti-mouse IgG conjugated with Alkaline Phosphatase Sigma Aldrich) (dilution 1:30,000) for 1 h at 37 °C in darkness. Colour development was performed using alkaline phosphatase substrate—pNPP, and the reaction was stopped with 5 mL of 5 M KOH. Absorbance was read at 405 nm with reference wavelength 605 nm in the microplate reader (Sunrise, Tecan). The measurement of 3-NT proteins was performed in three biological replicates, each in three technical replicates and results were expressed as pmol mg^−1^ protein.

### 2.10. Measurement of Oxidised RNA

Total RNA isolation was performed as described by Andryka-Dudek et al. [31]. The embryonic axes (100 mg) were ground in liquid nitrogen to a fine powder and placed in Eppendorf tubes, into each was added 1 mL of RNAzol^®^ RT (Sigma-Aldrich R4533) following the manufacturers’ instructions. Probes were vigorously mixed, and 0.4 mL of sterile, nuclease-free water (H_2_O_DEPC_) was added. After sample mixing and incubation at room temperature (15 min), centrifugation (15 min, 4 °C, 20,000× *g*) was performed. The supernatant (1 mL) was placed in the new Eppendorf tube, and 100% isopropanol was added (1:1). After incubation (15 min at room temperature) another centrifugation (22 °C, 10 min, 20,000× *g*) was done. The supernatant was removed, and the pellet was washed with 75% ethanol three times. After the last ethanol-washing step the pellet was air-dried and resuspended in 30 µL of H_2_O_DEPC_. The amount and quality of isolated RNA were measured using NANODROP 2000 Spectrophotometer (Thermo Scientific) at 230 nm, 260 nm and 280 nm.

RNA samples were digested according to [23] with some modifications, using nuclease P1 (Sigma-Aldrich N8630) and shrimp alkaline phosphatase (GE Healthcare UK E70092). RNA (10 µg) was treated with 1 U of nuclease P1. After 3 h of incubation at 37 °C, the pH of samples was adjusted to 7–8, then 1 U of shrimp alkaline phosphatase was added. Samples were placed at 37 °C for 1 h and boiled for 10 min.

Measurement of the content of oxidized RNA (8-oxyguanine, 8-OH-G) was performed using the DNA/RNA Oxidative Damage (High Sensitivity) ELISA kit (Cayman Chemical 589320) according to the manufacturer’s protocol. To the plate coated with secondary antibodies (goat anti-mouse IgG antibodies), 50 µL of the sample (containing 1 µg RNA) or standard attached to the kit were added. Then 50 µL of 8-hydroxy-2’deoxyguanosine-acetylcholinesterase conjugate (Tracer) and 50 µL of primary monoclonal antibodies were added. After 18 h of incubation at 4 °C, the solution was removed, and the plate was rinsed three times with 200 µL of wash buffer. Thereafter, 200 µL of Ellman’s reagent containing acetylcholine and 5,5′-thio-bis-(2-nitrobenzoic acid) was added. The plate was placed at 27 °C in the dark for 55 min. The absorbance was read at 412 nm in a microplate reader (Sunrise, Tecan). Measurement of oxidised RNA was performed in five biological replicates, each in two technical replicates and results were expressed as pg 8-OH-G µg^−1^ RNA.

### 2.11. Measurement of Nitrated RNA

Measurement of nitrated RNA (8-nitroguanine, 8-NO_2_-G) was performed using OxiSelect^TM^ Nitrosative DNA/RNA Damage ELISA kit (Cell Biolabs, Inc., San Diego, CA, USA) following the manufacturers’ instruction. RNA samples were prepared as described above.

Nunc Immuno Plate Maxisorp (Sigma) was coated with 100 µL of 8-NO_2_-G (1:400 dilution in PBS) per well, shaken for 45 min at room temperature, and incubated overnight at 4 °C. After incubation, 8-NO_2_-G was removed, and the plate was washed with PBS. Next, into the wells, assay diluent (200 µL per well) was added, and incubated for 1 h in darkness at room temperature. After removal of assay diluent, 50 µL of the sample was added into the well and incubated for 10 min shaking (VWR Thermal Shake Lite) at 27 °C. Next, antibodies (50 µL per well) anti-8-NO_2_-G (1:2000 diluted in AD) were added into the wells and incubated for 1 h at 27 °C. After this step, the plate was washed three times with washing buffer. Secondary antibodies 100 µL/well (1:1000 diluted in AD) were added into the wells and incubated for 1 h at 27 °C. Next, the plate was washed with washing buffer. Colour development was performed by adding the substrate solution into the wells (100 µL), and the reaction was stopped by adding 100 µL of stop solution. Absorbance was read at 450 nm (Sunrise, Tecan). 

The content of 8-NO_2_-G in RNA was calculated using a calibration curve. Measurements were performed in three biological replicates, each in three technical replicates and the results were expressed as pg 8-NO_2_-G µg^−1^ RNA.

### 2.12. Analysis of Genes Expression

The expression of genes was obtained in the axes of apple embryos using qPCR. Total RNA was isolated using RNAzol^®^ RT, following the manufacturer’s protocol. Total RNA (200 ng) was used for cDNA synthesis. The cDNA was obtained using RevertAid First Strand cDNA Synthesis Kit (Thermo Scientific, #K1622) in the total volume of 10 μL. The finished product was diluted 3.5 times. qPCR was carried out in a Bio-Rad CFX Connect™ Real-Time PCR Detection System. iTaq™ universal SYBR^®^ Green supermix (Bio-Rad, Hercules, CA, USA, #172–5124) was used for the reaction in the total volume of 12 μL (6 μL PCR supermix, 1 μL cDNA, 1 μL primer, 4 μL sterile H_2_O). Specific primers were designed based on nucleotide sequences available in the National Center for Biotechnological Information (NCBI) and the Genome Database for Rosaceae (https://www.rosaceae.org/ (accessed on 25 October 2019)) (Table S1). The expression levels were normalized using two reference genes and calculated using the method described by Vandesompele et al. [44] and Hellemans et al. [45]. The experiments were performed in three to five biological replicates, in three technical repetitions.

### 2.13. Statistics

The data were analysed using the Statistica13 Software. All data were obtained in at least 3 independent experiments with at least 2 repetitions each and presented as mean values ± SD. For gene expression analysis, the significant differences were evaluated using *t*-Student. For the results of other experiments, two-way ANOVA was used to determine the statistically significant difference between the means. The homogeneous groups were obtained by the post-hoc Tukey’s test.

## 3. Results

### 3.1. Accelerated Ageing of Apple Seeds Led to Alterations of the Biological Material

**Figure 2:**
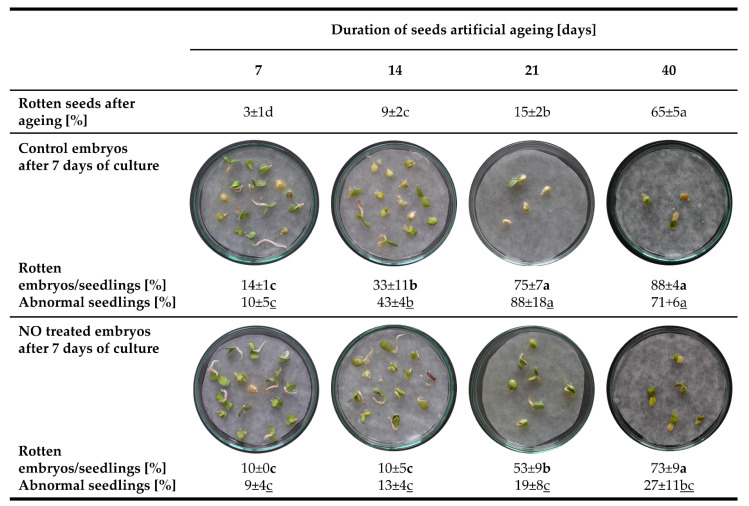
The number of rotten seeds determined after 7, 14, 21 or 40 days of accelerated ageing and the degenerative and morphological changes of embryos/seedlings after 7 days of the culture of embryos isolated from aged seeds (control) or embryos isolated from aged seeds and treated with NO. Values are the average of three independent experiments with 90 embryos in each (45 control and 45 NO treated). Homogenous groups were evaluated using Tukey’s test and signed as a–d (rotten seeds), **a**–**c** (rotten embryos/seedlings) and a–c (abnormal seedlings).

Prolonged artificial ageing of seeds increased the number of rotten seeds. After 21 days of seeds ageing, the tissue decomposition was characteristic for 15% of seeds, while after 40 days, the number of decayed seeds increased to 65% (Figure 2).

After 7, 14, 21 or 40 days of seeds ageing, the isolated embryos without visible tissue degradation were used as a control or treated with NO and placed in Petri dishes (15 embryos per dish) for 7 days (Figure 2). During the embryos culture, the increase in the number of rotten embryos/seedlings was observed in correlation with the prolongation of accelerated ageing. Embryos/seedlings with visible tissue disintegration were removed. After 7 days of ageing, the tissue of 14% of embryos/seedlings was damaged, while after 14 days of ageing the number of such embryos/seedlings was doubled. After 21 and 40 days of seeds ageing, around 80% of embryos/seedlings were completely disintegrated as the culture period was prolonged to 7 days (Figure 2). NO treatment of the embryos resulted in the 25% decrease in the number of rotten embryos/seedlings after 14 and 21 days of ageing. No beneficial effect of NO application was observed in the embryos isolated from seeds aged for 7 and 40 days compared to the adequate control (Figure 2).

Seedling abnormalities were defined as an asymmetric growth and greening of cotyledons [46]. The increased number of abnormal seedlings was noticed as the seeds artificial ageing was prolonged. NO fumigation declined the number of seedlings with morphological anomalies (Figure 2).

### 3.2. NO Improved Germination of the Embryos Isolated from Artificially Aged Seeds

**Figure 3:**
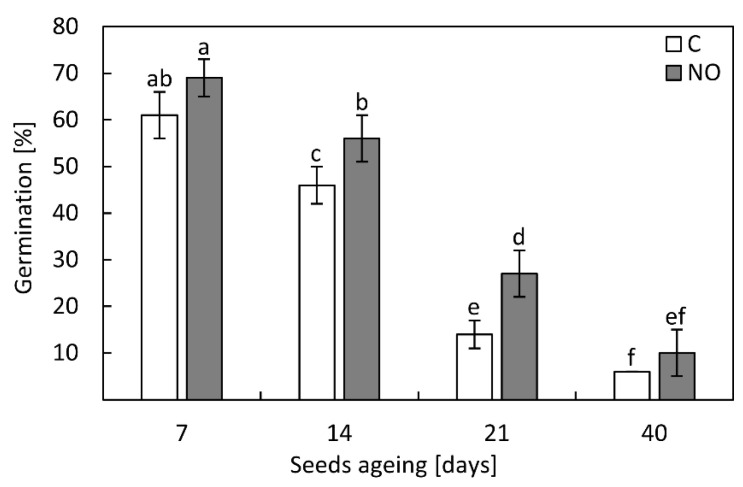
The germination rate of the apple embryos isolated from seeds subjected to accelerated ageing for 7, 14, 21 and 40 days (C) or the embryos isolated from aged seeds and treated with NO (NO). Values are the average of three independent experiments with 90 embryos in each (45 control and 45 NO treated). Different letters indicate significant differences obtained by Tukey’s test (*p* < 0.05).

Germination of the embryos isolated from seeds subjected to accelerated ageing for 7 days was 60% and no significant changes after NO treatment were observed (Figure 3). Prolongation of artificial ageing for the next 7 days resulted in the lowering number of germinating embryos. For these embryos, germination was 45% and increased to 56%, when the embryos were fumigated with NO. NO treatment also increased germination of the embryos isolated from seeds artificially aged for 21 days. After this period of ageing, germination of the control embryos reached only 14% but increased significantly (almost two-fold) after NO application. The worst germination rate was observed when seeds were subjected to accelerated ageing for 40 days. No differences in the embryos germination were noticed regardless of the embryos were treated with NO or not (Figure 3).

### 3.3. DNA Level Decreased as Accelerated Ageing Was Prolonged up to 40 Days

**Figure 4:**
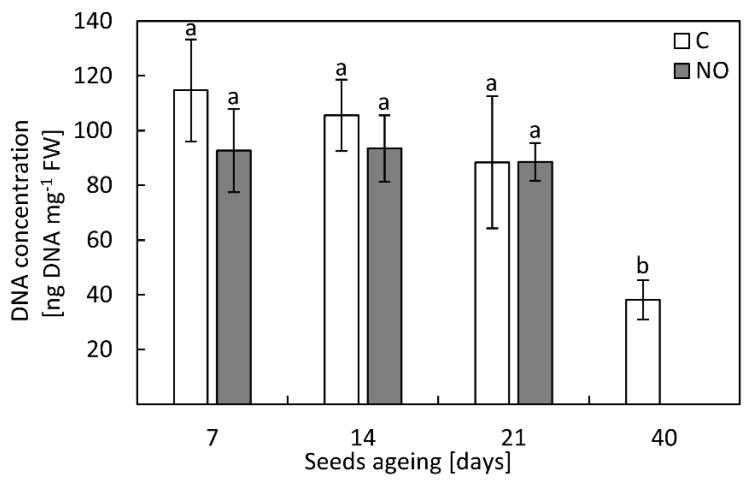
The DNA concentration in the axes of apple embryos isolated from seeds subjected to accelerated ageing for 7, 14, 21 and 40 days (C) or isolated from aged seeds and treated with NO (NO). Values are average ±SD of 3–4 independent repetitions. Different letters indicate significant differences determined by Tukey’s test (*p* < 0.05).

In the axes of control or NO-treated embryos isolated from seeds aged for 7, 14 and 21 days, DNA concentration was similar (Figure 4). DNA level decreased more than twice when artificial ageing of apple seeds was extended to 40 days.

### 3.4. NO Increased the Metabolic Activity in the Embryonic Axes of Aged Seeds

**Figure 5:**
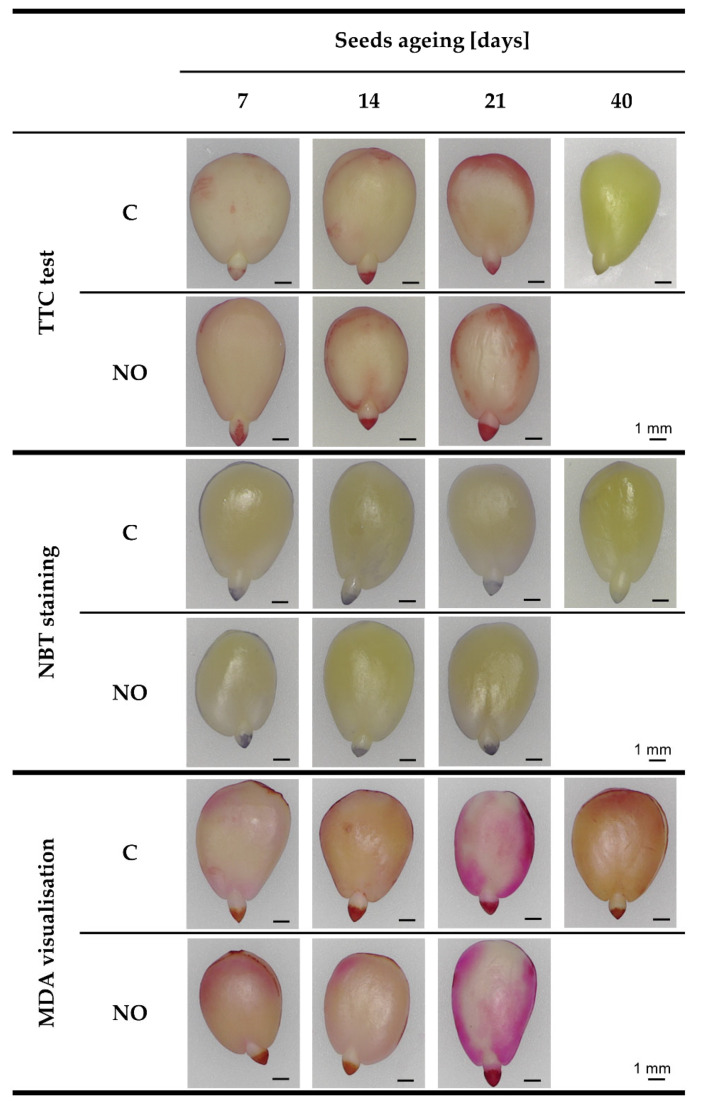
Visualization of staining of apple embryos isolated from seeds subjected to accelerated ageing for 7, 14, 21 and 40 days (C) or embryos isolated from aged seeds and treated with NO (NO), presenting the embryos viability (TTC test), in situ O_2_^•−^ (NBT staining) and MDA localisation. Pictures of representative embryos of 3–4 repetitions for each treatment were shown. Photos were taken using TAGARNO FHD TREND.

TTC staining allows to determinate the metabolic activity of the tissue, as TTC is reduced by mitochondrial succinate dehydrogenase to an insoluble formazan. The subtle coloration of the embryonic axes was characteristic for the embryos of control apple seeds aged for the shortest time (Figure 5). Among the embryos of artificially aged seeds (control), the highest pigments deposition was noticed after 14 days of ageing. After the longer period of ageing, red coloration of the embryonic axes was reduced (21 days of ageing) compared to the embryonic axes of seeds aged for 14 days or even undetectable (40 days of ageing). NO treatment increased the metabolic activity of the embryonic axes of seeds aged for 7 and 21 days (Figure 5).

### 3.5. NO Treatment Modified the Level of ROS in the Axes of Embryos Isolated from Aged Seeds

NBT staining did not show any changes in the ROS level in the axes of the control embryos isolated from seeds artificially aged for 7, 14 and 21 days (Figure 5). NO treatment enhanced ROS accumulation in the axes of embryos after 7 and 21 days of seeds ageing. After 40 days of accelerated ageing, the pigments deposition in the embryos was almost undetectable.

### 3.6. NO Treatment Changed the MDA Level in the Embryonic Axes of Aged Seeds

The deposition of pigments in the embryos isolated from seeds subjected to 7–14 days long artificial ageing was observed mainly in the embryonic axes (Figure 5). After 21 or 40 days of seeds ageing the coloration of cotyledons was also noticed. Based on colour intensity, among tissue non-treated with NO, embryos isolated from seeds aged for 7 days were characterized by the lowest level of MDA. Deposition of pigments in the axes of embryos of seeds aged for 14, 21 or 40 days was similar. NO fumigation did not affect the MDA level in the embryos isolated from seeds aged for 7, and 21 days, while after 14 days of seeds ageing, the reduced coloration of the embryonic axes was detected (Figure 5).

### 3.7. NO Increased the Antioxidant Capacity in the Embryonic Axes as the Ageing Procedure Was Prolonged to 21 Days

**Figure 6:**
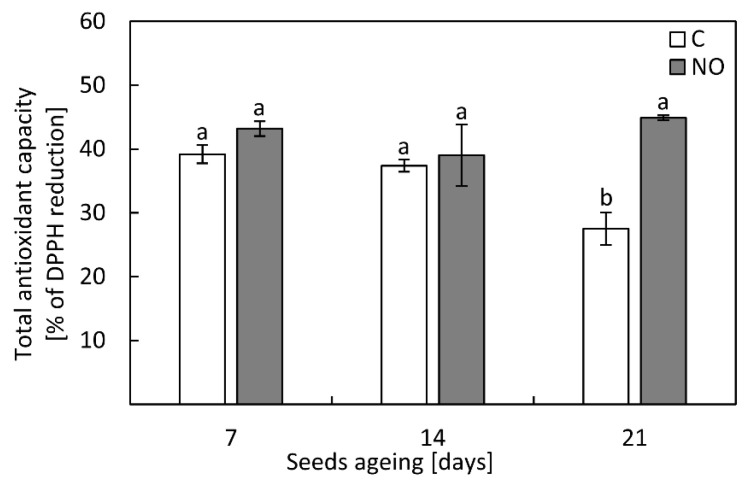
Total antioxidant capacity in the axes of apple embryos isolated from seeds subjected to accelerated ageing for 7, 14 and 21 days (C) or embryos isolated from aged seeds and treated with NO (NO). Values are average ±SD of 3-4 independent repetitions. Different letters indicate significant differences confirmed by Tukey’s test (*p* < 0.05).

After 7 or 14 days of seeds ageing, the antioxidant capacity was similar (around 40%) in the embryonic axes of control and NO-treated embryos (Figure 6). As the seeds were aged for 21 days, the antioxidant capacity decreased by 10%. After NO treatment, the value of the parameter increased 1.5-fold and reached the level observed in the axes of embryos isolated from seeds aged for 7 or 14 days (Figure 6).

### 3.8. NO Lowered the Levels of Nitrated and Oxidised RNA in the Embryonic Axes of Aged Seeds 

**Figure 7:**
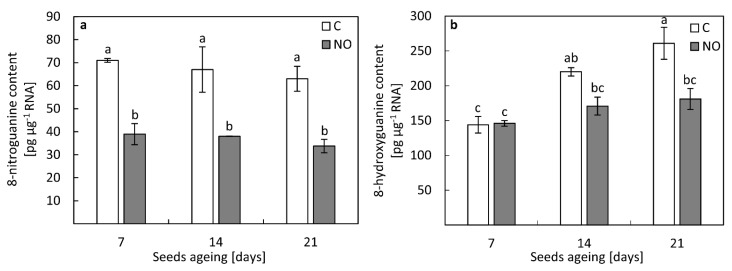
(**a**) The levels of nitrated and (**b**) oxidised RNA in the axes of apple embryos isolated from seeds subjected to accelerated ageing for 7, 14 and 21 days (C) or embryos isolated from aged seeds and treated with NO (NO). Values are average ±SD of 3–5 independent repetitions. Different letters indicate significant differences obtained by Tukey’s test (*p* < 0.05).

Regardless of the duration of seeds ageing, the level of 8-NO_2_-G in the embryonic axes was rather stable and reached around 70 pg µg^−1^ RNA (Figure 7a). NO treatment decreased the level of nitrated RNA almost twice after 7, 14 and 21 days of seeds ageing compared to the control. 

The extension of the duration of seeds ageing resulted in the gradual increase in the level of oxidised RNA (Figure 7b). After 14 days of seeds ageing, in the embryonic axes the content of 8-OH-G enhanced by 50%, while after 21 days of ageing, increased by 80% compared to the embryonic axes of seeds aged for 7 days. NO application did not change the level of modified RNA after 7 days of seeds ageing. Fumigation of the embryos isolated from seeds subjected to accelerated ageing for 14 or 21 days with NO decreased the level of oxidised RNA, by 20% and 30%, respectively (Figure 7b).

### 3.9. NO Modified the Content of Nitrated Proteins Only in the Embryonic Axes of Seed Aged for 7 Days 

In the embryonic axes, the level of nitrated protein was around 100 pmol mg^−1^ protein regardless of the duration of seeds ageing (Table 1). Changes in the content of these proteins were noticed only in the axes of NO-treated embryos, isolated from seeds artificially aged for 7 days. The level of modified proteins decreased by 31% compared to the control (Table 1). 

### 3.10. NO Did Not Affect the Content of Oxidized Proteins in the Embryonic Axes of Aged Seeds 

The level of carbonyl groups in the axes of embryos isolated from aged seed did not change as the duration of seed ageing was extended. NO treatment of the apple embryos did not impact the content of oxidized proteins regardless of the time of seeds ageing (Table 1). 

### 3.11. NO Changed the Expression of Genes Encoding Enzymes of ROS Metabolism in the Embryonic Axes of Aged Seeds

**Figure 8:**
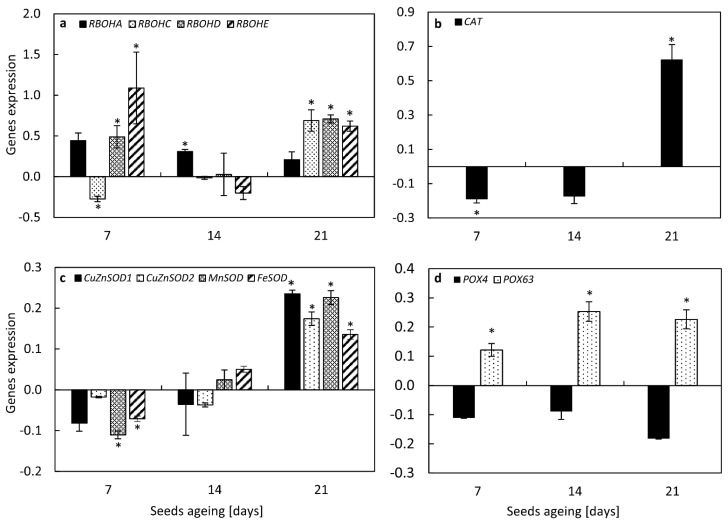
The relative transcript levels of genes encoding RBOH homologues (**a**) *RBOHA*, *RBOHC, RBOHD*, *RBOHE*, (**b**) *CAT*, (**c**) *CuZnSOD1*, *CuZnSOD2*, *MnSOD*, *FeSOD* and (**d**) *POX4*, *POX63* in the axes of apple seeds subjected to accelerated ageing for 7, 14, and 21 days, treated with NO, analyzed by qRT-PCR as described in the section of Material and Methods. Values are average ±SD of 3–5 repetitions. Asterisks indicate significant differences between the treated sample compared to the adequate control sample, obtained by the Student test (*p* < 0.05).

After 7 days of accelerated ageing, NO treatment increased the expression of genes encoding RBOHD and E homologs (Figure 8a). In the axes of embryos of those seeds, after NO fumigation, the decrease in *RBOHC* and no changes in *RBOHA* expression levels were noticed. After 14 days of seeds artificial ageing, as a result of NO application, only *RBOHA* was upregulated. In the axes of apple embryos isolated from seeds aged for 21 days, NO treatment led to the upregulation of *RBOHC, D* and *E* (Figure 8a), while the expression of *RBOHA* did not differ.

NO treatment of the embryos isolated from seeds aged for 7 days resulted in the decrease in *CAT* expression (Figure 8b). The transcription level of this gene increased in the axes of seeds subjected to accelerated ageing for 21 days. The same expression level of *CAT* was characteristic for the axes of NO-fumigated and non-treated embryos isolated from seeds aged for 14 days (Figure 8b).

Application of gaseous NO to the embryos isolated from seeds artificially aged for 7 days resulted in the decrease in *MnSOD* and *FeSOD* transcript levels (Figure 8c). In the embryonic axes of those seeds, no significant changes in the expression of genes encoding *CuZnSOD* isoforms were detected. The expression of all tested genes did not differ in the axes of treated embryos, isolated from seeds aged for 14 days. After 21 days of accelerated ageing, the embryos fumigation with NO led to the upregulation of *CuZnSOD1* and 2, *MnSOD* and *FeSOD* (Figure 8c).

The changes in the *POX4* transcript level were characteristic only for the axes of apple embryos isolated from seeds aged for 21 days, treated with NO (Figure 8d). In this tissue, the decrease in expression of *POX4* was noticed. NO application enhanced the transcript level of *POX63* regardless of the time of seeds ageing.

## 4. Discussion

Apple seeds are characterised by a deep, embryonic dormancy. Dormancy loss of apple seeds occurs during cold stratification [39]. Contrary, warm stratification at 25 °C led to seeds mortal deterioration [11]. In our experiments, apple seeds were subjected to accelerated ageing at 33 °C, and 65% humidity. As a result of this procedure, especially when it was carried out longer than 14 days, the visible symptoms of embryo ageing were noted (Figure 2). After 40 days of artificial ageing around 65% of seeds were disintegrated, and among the rest seeds, more than 80% of isolated embryos were rotten during the first 7 days of the culture (Figure 2). The decreased viability of these embryos was confirmed using the TTC staining test (Figure 5).

The cold stratification conditions favour metabolic processes that do not happen at the higher temperature. For example, cyanogenesis resulting in the liberation of hydrogen cyanide (HCN), which could stimulate NO generation [46], is observed only in apple seeds stratified at 5 °C [39,47]. Fumigation of dormant apple embryos with HCN resulted in the increased NO emission from the embryonic axes leading to the dormancy removal and high germination rate [46]. As the conditions of higher temperature and higher relative humidity during warm stratification of apple seeds interfere with NO production [11], some questions were raised, e.g., whether NO supplementation of embryos isolated from *orthodox* seeds imbibed at high temperature and high humidity may delay the cellular deterioration, and why is the NO optimal level so important for seed viability?

The beneficial effects of NO fumigation on the support of viability in artificially aged elm (*Ulmus pumila* L.) and oat (*Avena sativa* L.) seeds were confirmed [48,49]. Ageing of elm seeds was induced by CDT (37 °C and 100% relative humidity) [49]. Prolonged CDT of elm seeds was accompanied by a decrease in endogenous NO content. Supplementation of sodium nitroprusside (SNP), as NO donor, into the culture medium before CDT, maintained the high seed vigour, and improved germination rate [49]. For oat seeds, the increased seed viability was achieved when SNP was applied after seeds ageing [48].

In our work, the most visible, positive effect of short-term NO application, on the maintenance of apple embryos viability, was observed after 14 and 21 days of seeds accelerated ageing. The germination rate of NO-treated embryos was higher, and developing seedlings exhibited less morphological abnormalities than the control (Figure 2 and Figure 3). Previously, we have demonstrated the equal greening of cotyledons of abnormal apple seedlings after NO supplementation [50], proving an important mechanism of switching from heterotrophy to autotrophy. Similarly, NO stabilized photosynthetic pigments in cotyledons of soybean (*Glycine max* L.), suggesting its role in ageing delay [51].

Overaccumulation of H_2_O_2_ was noticed in axes of aged sunflower (*Helianthus annuus* L.) seeds [25]. The higher H_2_O_2_ content was also observed in oat seeds subjected to artificial ageing for 16 and 32 days [52], and in aged soybean seeds [53]. Low vigour of artificially aged oat seeds was linked to the accumulation of H_2_O_2_ in mitochondria, while the O_2_^•−^ content did not differ compared to non-aged seeds [48]. Visualization of free radicals (O_2_^•−^) in the apple embryonic axes also showed a steady-state level during accelerated ageing up to 21 days (Figure 5). In soybean seeds subjected to artificial ageing, the highest ROS content (O_2_^•−^ and H_2_O_2_) was observed after 18 days. While after 41 days of ageing ROS level decreased (H_2_O_2_) or did not differ (O_2_^•−^) compared to non-aged seeds [53]. We noted the similar tendency for the apple embryos isolated from seeds aged for 40 days (Figure 5). NO treatment of embryos isolated from apple seeds aged for 7 and 21 days increased the level of free radicals in the embryonic axes. Dormancy release of viable apple embryos by NO was accompanied by increased free radicals in embryonic axes [26]. Free radicals generation is partly due to the activity of RBOH [24]. During seed germination free radicals, produced also by RBOH, take part in cell wall loosening, hypocotyl elongation, and malting processes, which have been demonstrated for barley (*Hordeum vulgare* L.) seeds [54]. As was proposed, AtRBOHB is a regulator of the after-ripening of Arabidopsis seeds via modulation of carbonylated proteins level [24]. On the other hand, *RBOHD*, *RBOHE*, and *RBOHF* were identified as negative regulators of Arabidopsis seed longevity [55]. We observed, the increase in *RBOHC, D, E* transcript levels in the axes of NO-treated embryos after 21 days of artificial ageing (Figure 8a). Upregulation of only *RBOHA* was characteristic for embryos fumigated with NO after 14 days of ageing. In the axes of embryos of seeds aged for 7 days, fumigated with NO, transcript levels of two homologs of RBOH (*RBOHD, E*) were elevated. This indicates a diverse NO action, depending on the ageing progression of apple seeds.

One week-long accelerated ageing resulted in activation of apple seeds metabolism. At this time, when seeds were imbibed in wet sand and temperature above 30 °C stimulation of enzymatic activities, typical for seeds at germination probably occurred. Embryos treatment with NO additionally strengthened metabolic activity of the tissue, replacing HCN liberation. Accumulation of O_2_^•−^ correlated to decreased transcript levels of *SOD, CAT* and high transcript levels of *RBOH.* We suppose that possibly non-enzymatic, ROS-dependent production of ethylene (a hormone that stimulates apple embryos germination [46]) was initiated, leading to faster germination of the embryos and growth of the seedlings. Whereas, 40 days of artificial ageing of apple seeds resulted in the loss of seed vigour, as was demonstrated using TTC staining (Figure 5). Short-term fumigation with NO of embryos isolated from seeds aged for 40 days did not lead to recovery, and even more severe damages were observed (data not shown). Thus, we assume that after a long-lasting seeds ageing process NO acts as an additive stress factor.

**Figure 9:**
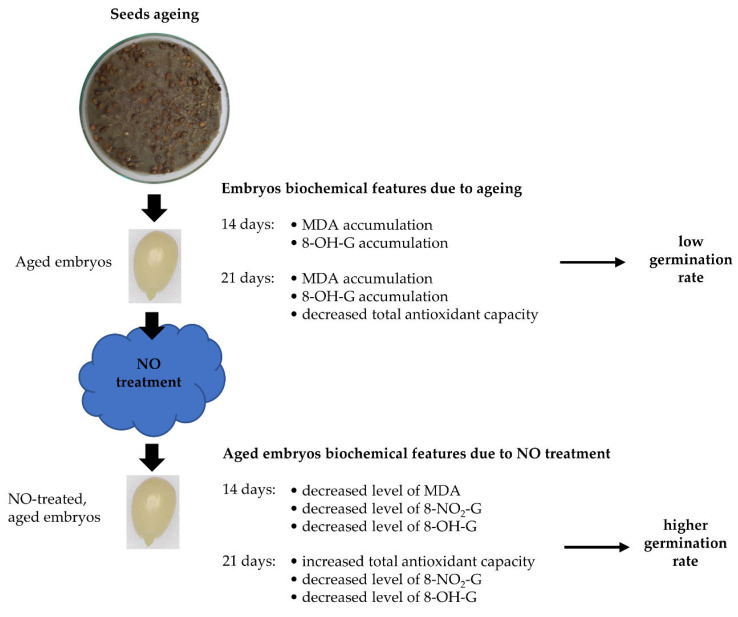
The simplified model of alterations in some biochemical features of the embryos isolated from apple seeds undergoing aging for 14 or 21 days. These embryos are characterized by the low germinability. The parameters are chosen in comparison to the embryos isolated from seeds aged for 7 days. NO beneficial effect on the embryos isolated from aged seeds refers to modification of identified biochemical attributes, leading to the restoration of high germination rate (after 14 days) or the prevention of the drastic decrease in germination rate (after 21 days). The features are chosen based on the difference of the parameters values between the aged and NO-treated aged embryos.

The increased level of MDA connected with prolonged artificial ageing of seeds was demonstrated for soybean [53], grugru palm (*Acrocomia aculeata* (Jacq.)) [56], and in naturally aged tobacco (*Nicotiana tabaccum* L.) seeds [57]. Aldehydes, including MDA visualisation, demonstrated the increase in the level of lipid oxidation products (observed also in cotyledons) with the progression of apple embryos artificial ageing (Figure 5 and Figure 9). However, NO fumigation did not lower the intensity of colour development in embryos tissue, suggesting the role of NO in the protection of other than lipids cellular structures or the participation of oxidised lipids in signal transduction, as some derivatives of lipids may play signalling function [58].

According to the free radical theory of ageing, the increase in ROS content is linked to the lower activity/level of antioxidants. This was demonstrated for artificially aged soybeans seeds, the activity of basic antioxidants (SOD, CAT, ascorbic peroxidase) decreased [53]. Contrarily, in oat seeds aged for 32 days, the increase in SOD and CAT activity was noted [52]. In the apple embryonic axes isolated from seeds subjected to accelerated ageing the decrease in DPPH reduction was observed after 21 days of the experiment. Short term fumigation with NO of embryos isolated from such aged apple seeds restored antioxidant capacity (Figure 6), suggesting the contribution of low-molecular weight antioxidants in ROS scavenging. At the same time point of ageing, we observed an increase in the transcript levels of *SOD*, *CAT* and *POX63* in the axes of embryos shortly fumigated with NO (Figure 8b–d). The beneficial effect of NO on the activity of enzymatic antioxidants in seeds was reported before [26,48,49]. Priming of aged sunflower seeds was linked to the higher level of *CAT* [25]. The authors proposed that the lowering of *CAT* transcript level in aged sunflower seeds was mostly due to the degradation of oxidized RNA [25]. For apple seeds, the lower DNA content was noticed only in embryonic axes isolated from seeds subjected to artificial ageing for 40 days (Figure 4). The presence of nitrated RNA was confirmed in dormant and non-dormant apple embryos [31]. Maturation of seeds is accompanied by the accumulation of mRNA required for the activation of metabolism during the early phase of imbibition [23,59]. This stored mRNA undergoes some modifications, e.g., oxidation [23,59,60]. Oxidation of specific mRNA in sunflower seeds was a “switch” from dormancy to germination stage [23]. In the axes of apple embryos isolated from seeds subjected to accelerated ageing (7–21 days) the amount of 8-NO_2_-G in the total RNA was higher compared to NO fumigated embryos (Figure 7a). Prolonged apple seeds ageing was also related to the high level of oxidized RNA in the embryonic axes. NO application after seeds ageing lowered the oxidative/nitrosative damages of RNA. This indicates that NO, acting as a remedy, could switch on the repair of ROS induced cellular deteriorations (Figure 9).

In apple seeds subjected to artificial ageing for 14 and 21 days, we observed the similar level of total carbonylated proteins regardless of the embryos were exposed to NO (Table 1). In opposite, in our previous experiments, prolonged warm stratified apple seeds were characterised by the higher H_2_O_2_ content and the elevated level of CO groups in proteins [11]. Ageing of Arabidopsis seeds was associated with the increase in the content of carbonylated proteins [1,8]. Extended storage of beech (*Fagus sylvatica* L.) seeds was also connected with the gradual elevation in the level of such modified proteins [61]. Among proteins with CO groups, the authors identified proteins involved in desiccation tolerance or cell division. It may point that measurement of the quantity of protein CO groups level is not adequate to determine the anti-ageing NO action. The quality of proteins with CO groups needs to be examined.

NO-derived protein modification concerned some metabolically important proteins potentially engaged in seed deterioration, as was suggested by He et al. [49]. At the early stages of ageing of elm seeds they observed the high amount of *S*-nitrosylated proteins, that lowered during ageing progression [49]. In our study, we investigated the level of nitrated proteins in the apple embryonic axes. Similarly, for oxidized proteins, the total level of 3-NT containing proteins in control (artificially aged only) and NO treated embryos was similar when seeds ageing lasted 14 days or longer (Table 1). After 7 days of accelerated ageing (not related to the deterioration process), NO application resulted in the decline of nitrated proteins content, corresponding with our previous data. We demonstrated that the nitrated proteins level decreased during germination of apple embryos, dormancy of which was removed by NO fumigation [37]. Moreover, we proposed that most nitrated proteins belong to the storage proteins utilized along with the time of imbibition. As protein nitration is a highly selective process, which modifies protein function [62] we conclude that NO-mediated nitration of proteins in seeds is an important mode of action of this compound. To make this statement stronger, the analysis of nitrated proteins need to be extended and should include the identification of particular proteins.

## 5. Conclusions

Summarizing our results, we have demonstrated the remedial role of NO in oxidative remodelling after seeds artificial ageing (Figure 9, Table 2). The answer to the question about the beneficial role of NO in the restoration of the viability of the aged apple seeds is affirmative, but only for the seeds subjected to artificial ageing for no longer than 21 days. After-ageing NO fumigation of apple embryos stimulated germination and increased ROS level probably due to the elevated expression of *RBOH*. We can assume that putative stimulation of RBOH activity following upregulation of RBOH encoding genes allowed the embryos to achieve ROS content located in-between the rate of “oxidative window” [7] typical for seeds at germination state. Stimulation of total antioxidant capacity by NO fumigation was accompanied by increased transcript levels of genes encoding enzymatic antioxidants SOD and CAT, that could protect the embryonic tissue against overaccumulation of destructive ROS. The more, post-aged NO application, in concentration referring to the “nitrosative door” [26], diminished nitro-oxidative modification of RNA, possibly ensuring the synthesis of correct proteins.

## Figures and Tables

**Table 1:** The levels of protein nitration and protein carbonylation in the axes of apple embryos isolated from seeds subjected to accelerated ageing for 7, 14 and 21 days (C) or embryos isolated from aged seeds and treated with NO (NO). Values are average ±SD of 3 independent repetitions. Different letters indicate significant differences obtained by Tukey’s test (*p* < 0.05).

	Nitrated Proteins [pmol mg^−1^ Protein]		Carbonylated Proteins [nmol mg^−1^ Protein]	
Seeds ageing [days]	**C**	**NO**	**C**	**NO**
7	94.5 ± 9 a	60.6 ± 2 b	0.67 ± 0.02 a	0.59 ± 0.07 a
14	90.4 ± 14 a	103.6 ± 19 a	0.59 ± 0.10 a	0.51 ± 0.09 a
21	106.3 ± 19 a	99.1 ± 12 a	0.52 ± 0.03 a	0.51 ± 0.03 a

**Table 2:** Post-ageing NO action in the apple embryos. The changes of the rate of the parameters analyzed in the experiment in which embryos isolated from seeds subjected to 21 days of accelerated ageing were fumigated with NO.

Parameter	Effect after NO Fumigation
Germination	↑
Metabolic activity	─
ROS level	↑
RBOH transcript levels	↑
MDA	─
RNA oxidation	↓
RNA nitration	↓
Protein oxidation	─
Protein nitration	─
Total antioxidant capacity	↑
SOD transcript levels CAT transcript levels	↑ ↑

Abbreviation: ↑ for increase, ↓ for decrease and ─ for no shift of the parameter in comparison to the control.

## Data Availability

The data presented in this study are available on request from the corresponding author. The data is not publicly available because it is not stored on the cloud storage or file storage services.

## Supplementary Materials

The following is available online at https://www.mdpi.com/article/10.3390/antiox11010070/s1 Table S1: list of primers.
