# Supplementary material for: Nitric Oxide as a Remedy against Oxidative Damages in Apple Seeds Undergoing Accelerated Ageing
Source: Antioxidants (Basel). 2021 Dec 28;11(1):70. doi: 10.3390/antiox11010070 (PMC8772863; doi:10.3390/antiox11010070)
Supplement: Supplementary file 1 [file antioxidants-11-00070-s001.zip › antioxidants-1526356-supplementary.pdf]

Table S1. List of primers

Gene symbol	Primer sequence 5′-3′	Encoded Protein	Gene ID (NCBI/ GDR)
MdRBOHA	F:GTGCTGTTGCCTGTTTGTAGAA<br>R:TTGTGGAAGTTGATGTTGTCG	respiratory burst oxidase homolog A	MD14G1113700<br>MD06G1093000
MdRBOHC	F: AAAGGGTTGGAGAAGAGCCG<br>R: GTTCGATCAAACCGCCTTCG	respiratory burst oxidase homolog C	XM_008377211.3
MdRBOHD	F:CACCAGCACTAACAAAGGACCTCA<br>R:TCTCTTTGTGGAAGTCGAACTTC	respiratory burst oxidase homolog D	MD16G1145100
MdRBOHE	F: CGCACCGCTATCCTCAGATT<br>R: TCCAGTCGTGCCTTCAGTTC	respiratory burst oxidase homolog E	XM_008339593.3
MdPOX4	F: ATAAAGTGGAACTTCACCA<br>R: CGTACCGTTCAATGACATG	peroxidase 4	MD11G1289300<br>MD03G1268500
MdPOX63	F: CACACCATCGGGTTCTCCCA<br>R: CCGACTTGCTGTAGTTGTAGA	peroxidase 63	MD09G1039100<br>MD17G1040100
MdCuZnSOD1	F: GGAAACATCACTGCTGGGGA<br>R:TTGCCAAGGTCATCAGGGTC	copper zinc superoxide dismutase 1	XM_008372433.3
MdCuZnSOD2	F:CACTGAGTGGACCTGATTCCAT<br>R: TGACTTAAGGCCAATGATACCACA	copper zinc superoxide dismutase 2	XM_008388606.3
MdFeSOD	F: GAGAGAAGAGAGACGACTTCAG<br>R: CCACGTACTTTCCTCTGTCA	iron superoxide dismutase	XM_008363690.3
MdMnSOD	F: GTCAAGGTGGAGGTGAGTCC<br>R: CAAGCCACACCCATCCAGAA	manganese superoxide dismutase	XM_008371134.3
MdCAT	F:CATCTGGTGGAGAAACTCGCCAACT<br>R:CACAATGACAGGTGTCTGAACTCCAG	catalase	XM_029104354.1
MdSAR1	F: TTGATTTGGGCGGGCATCAGATTG<br>R: TCATCAGAGAGGAGAGCATCCAGC	small GTP-binding protein	MD04G1194800
MdPDI	F: TGCTGTACACAGCCAACGAT<br>R: CATCTTTAGCGGCGTTATCCTTG	protein disulfide isomerase	XM_008344461.2
